# Supplementary material for: Tracking DOT1L methyltransferase activity by stable isotope labelling using a selective synthetic co-factor
Source: Commun Chem. 2024 Jun 27;7:145. doi: 10.1038/s42004-024-01227-x (PMC11211345; doi:10.1038/s42004-024-01227-x)
Supplement: Supplementary file 2 — Supplementary Information [file 42004_2024_1227_MOESM2_ESM.pdf]

Supplementary Information

Tracking DOT1L Methyltransferase Activity by Stable Isotope Labelling Using a Selective Synthetic Co-Factor

Nicole Trainor,[‡a] Harry J. Whitwell,[‡b,c] Beatriz Jiménez,[b,c] Katie Addison,[a] Emily Leonidou,[a,d] Peter A. DiMaggio[d] and Matthew J. Fuchter*[a]

*Email: m.fuchter@imperial.ac.uk

‡These authors contributed equally

[a]Department of Chemistry, Molecular Sciences Research Hub, Imperial College London, White City Campus, 82 Wood Lane, London W12 OBZ, United Kingdom

[b]National Phenome Centre and Imperial Clinical Phenotyping Centre, Department of Metabolism, Digestion and Reproduction, IRDB, Building Imperial College London, W12 ONN, United Kingdom

[c]Section of Bioanalytical Chemistry, Division of Systems Medicine, Department of Metabolism, Digestion and Reproduction, Sir Alexander Fleming Building, Imperial College London, SW7 2AZ, United Kingdom

[d]Department of Chemical Engineering, Imperial College London, South Kensington Campus, London SW7 2AZ, United Kingdom

Contents

Supplementary Methods – page 2

Supplementary Tables 1-6 – page 4

Supplementary Figures 1-6 – page 8

Chemical synthesis – page 14

Spectra for 7 and 8: Supplementary Figures 7-11 – page 19

Supplementary References – page 22

Methods

In vitro methylation reactions

The methyltransferase enzymes SETD7, EHMT1, SETD2, SUB420H2 were kindly donated by Prof. M. Vedadi (University of Toronto). The catalytically competent recombinant DOT1L truncated protein was purchased from Life Technologies Ltd (11552HNCE250) and HeLa derived nucleosomes from AMSBIO (52039). Reactions conditions were performed as described here[1] (see also Supplementary Table 2).

Cell Lysate Methylation Reactions

HEK293T cells were cultured in T75 flasks with DMEM supplemented with 10% fetal bovine serum in a humidified atmosphere at 37 °C with 5% CO_2. Cells were maintained at 50-80% confluency and >95% viability. Prior to cell lysis, cells were treated with 0.1 µM of the DOT1L inhibitor EPZ5676 (Stratech, S7062) for 4 days. Lysates were prepared by washing cells 3x with chilled PBS and recovered from the surface of a cell culture flask with 1 mL Trypsin/EDTA solution. Cells were centrifuged for 5 minutes at 300 xg at 4 °C to remove the trypsin and then suspended in 0.1 mL 50 mM Tris-HCl, 5 mM $MgCl_2$, 4 mM DTT, 0.1% NP-40, 0.2 mM PMSF and 10 units/mL DNase and left on ice for 5 minutes. After douncing cells (60 strokes on ice), the lysate was centrifuged at 18000 xg for 10 minutes (4 °C) to remove debris and the quantity of recovered proteins was determined using a Protein Assay 2 kit (Biorad) as per the manufacturer's instructions. Reactions with DOT1L were performed for 3 hours at 37 °C in 500 µL 50 mM Tris, 5 mM $MgCl_2$ and 4 mM DTT, as described in Supplementary Table 3. Reactions were quenched by adding 4x the sample volume (2 mL) of ice-cold methanol and immediately performing chloroform-methanol precipitation. Precipitates were dissolved in 50 µL 8 M urea in 100 mM ammonium bicarbonate and reduced with 10 mM tris(2-carboxyethyl)phosphine (TCEP) for 30 minutes at 37 °C. After alkylating with 15 mM iodoacetamide (30 minutes, room temperature in the dark), the total volume was increased to 350 µL with 100 mM ammonium bicarbonate and the proteins were digested with sequencing grade modified trypsin/LysC (Promega) at 1:50 (protease:protein) overnight. Digested lysates were desalted and fractionated into 8 using Pierce high pH fractionation kit as per the manufacturer's instructions and fully dried at room temperature in a vacuum centrifuge.

LC-MS

Each sample was dissolved in 20 µL 0.1% formic acid (FA) by titration followed by sonicating for 10 minutes. After centrifuging for 2 minutes at 13,000 x g, samples were transferred to glass sample vials. 5 µL was injected onto a 2 cm x 75 µm Acclaim Pepmap 100 trap column for 3 minutes, before being separated on an 15 cmx 75 µm Acclaim Pepmap RSLC column over 140 minutes, starting in 92% (5% dimethylsulfoxide (DMSO), 0.1% FA), ramping to 45% (5% DMSO, 0.1% FA, 75% acetonitrile) over 110 minutes using a Ultimate 3000 HPLC (Thermo Scientific) interfaced with an IT-FT mass spectrometry (Orbitrap Velos, Thermo Scientific) via a nano-ESI source. Mass spectrometry was performed using Top 10 CID, over a range of 200-2000 *mz* at 60k resolution. Singly charged parent ions were excluded from MSMS acquisition and a 20 second exclusion list (with repeat measurements within 20 seconds enabled) was enabled. Fragmentation was performed by CID at a normalized energy of 30.

Database Searching

Database searching was conducted in proteome discoverer 2.2 (Thermo Scientific) using the Mascot search engine (Matric Science), searching the Swissprot human reference proteome (Uniprot, downloaded April 2018). Carbamidomethylation was set as a fixed modification with oxidized methionine, and heavy/light methylation (mono, di, tri)-K and (mono, di)-R were set as variable

modifications. Percolator was used to rescore peptides with FDR filtering set at 1% (strict) and 5% (relaxed).

MS2 Matching

MS2 spectra that gave rise to a peptide spectral match were searched against MS2 spectra arising from parent ions with a mass shift corresponding to heavy/light labeling (i.e. ~4 Da shift) using an in-house script. Briefly, mass spectrometry files were converted to mzXML using default setting in MSConvert[2] and read into R using the mzR package. For each MS2 with a PSM (from Mascot database searching), peak lists were extracted for MS2 scans arising from parent ions with a 4Da shift. Peak lists were exported and compared for matching and complementary ions as described for HiLight-PTM[3], using a tolerance of 0.5 and 0.8 *mz* for matching the same and complementary-mass shift ions respectively. Pearson's product moment correlation coefficient was then calculated in R for the intensity of the matched and complementary mass-shifted ions.

Quantitative kinetic analysis of H3K79 methylation by DOT1L with $^{13}CD_3$ co-factor analogues

The K79 peptide standard used for the following quantitative kinetic analyses (Supplementary Figure 2 and 3) was purchased from the AQUA Peptide laboratory of Sigma Aldrich. The procedures for the preparation of histone peptides for MS analysis were previously published by Lin and Garcia[4].

Reactions were carried out at 37 °C in KMT buffer (7.5 mM Tris.HCl pH 8.8, 10 mM $MgCl_2$) with a total assay volume of 200 µL. Other components were present in the following amounts:

4 mM DTT
10 µg HeLa-derived nucleosomes
50 nM DOT1L

For the time course study, at the required interval (15, 30, 45 or 60 minutes) reactions were performed using 100 µM $^{13}CD_3$SAM or $^{13}CD_3$BrSAM. For the titration study, reactions were performed for 15 minutes, using concentrations between 100 nM and 1 mM of $^{13}CD_3$SAM or $^{13}CD_3$BrSAM. Reactions were quenched with the addition of 1 mM cold SAH (dissolved in 99% H_2O, 1% FA). After quenching, samples were subsequently dried using a speedvac and then subjected to preparation for MS analysis.

Three replicates per data point were obtained and absolute quantitative analysis was carried out using the addition of 2 pmol of a K79un-containing peptide standard bearing a heavy labeled arginine residue at the C-terminus. The peptide standard was calibrated by addition to unreacted nucleosomes prepared for MS in a manner analogous to those obtained from reaction conditions (see Supplementary Figure 4-5). Only the unmodified and mono-methyl (light and heavy) states were investigated; the error associated with excluding heavy di-methyl signals was considered to be negligible as the total proportion of di-methylated states in a sample reaches no larger than 2.6% ($^{13}CD_3$SAM) and 1% ($^{13}CD_3$BrSAM) across the 60 minute time frame.

The following criteria were applied to assess MS sample quality:

- Signal intensity of the peak of interest (LC) should be greater than 1×10^5 arbitrary units
- The peak of interest (MS) should appear in the absence of interference from impurities

Supplementary Tables

**SAM analogue**	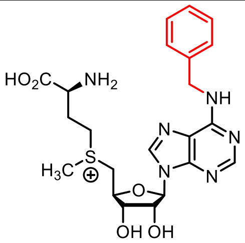	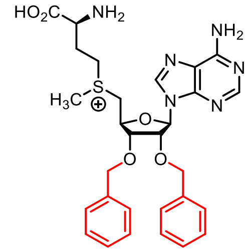	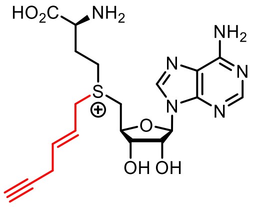	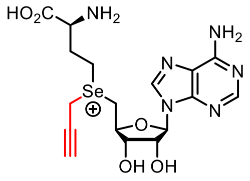
**Modification**	N^6-benzyl SAM[5]	2',3'-dibenzyl SAM[6]	Sulfonium-alkyl SAM[7]	Propargylic Se-adenosyl-l-selenomethionine (ProSeAM)[8, 9]
**PMT, Mutation**	Rmt1 E117G	vSET L116A	G9a Y1154A	Various WT PMTs
**PMT Substrate**	Arginine residues	Histone H3K27	Histone H3K9/H3K27	Various protein and RNA substrates
**Substrate target assay**	Synthetic Prmt1 Peptide	H3 Peptide (13-33)	Histone peptides, cells	Cell lysates

Supplementary Table 1: Summary of previous studies of protein methyltransferase (PMT) activities using the bump-and-hole approach.

Protein	UniProt Accession	Target	[SAM] (µM)	[Substrate] (µM)	[Enzyme] (nM)	Buffer	pH	[DTT] (mM)	Triton-X100 (%)
SETD7	Q8WTS6	H3K4	1000	1	1000	50 mM Tris-HCl	8	5	0.01
EHMT1/GLP/G9A	Q9H9B1	H3K9	1000	1	1000	25 mM sodium phosphate	8	0	0.01
SETD2	Q9BYW2	H3K36	13.3	1	306.5	50 mM Tris-HCl	9	5	0.01
SUV420H2	Q86Y97	H4K20	1000	1	1000	50 mM Tris-HCl	8	5	0.01
DOT1L	Q8TEK3	H3K79	1000	1	1000	50 mM Tris-HCl + 5 mM $MgCl_2$	8.8	4	0

Supplementary Table 2: Summary of the conditions used for nucleosome labelling experiments. All reactions were performed at 37 °C for one hour with a total reaction volume of 0.2 mL.

Sample:	Negative	Positive	A (90%)	B (80%)	C (70%)	D (60%)
Lysate	50µg	50µg	50µg	50µg	50µg	50µg
Nucleosomes	10µg	10µg	2µg	2µg	2µg	2µg
DOT1L	0µM	0.4µM	0.4µM	0.4µM	0.4µM	0.4µM
$SAM^{(CH3)}$	0.1mM	0.1mM	0.1mM	0.2mM	0.3mM	0.4mM
$BrSAM^{(13CD3)}$	0.9mM	0.9mM	0.9mM	0.8mM	0.7mM	0.6mM

Supplementary Table 3: Reagent quantities for DOT1L reactions in cell lysate. Percentages in sample names relate to the proportion of $^{13}CD_3$-BrSAM as the methyl donor.

expt_mz	expt_intensity	ms2seq	ms2type	theory_mz	dDa	dPPM
371.6833	32	EIAQDFkTDL	[a10]3+	371.8947	-0.211	-567.3648867
215.1833	2	EI	[a2]1+	215.139	0.044	204.5189199
456.7778	8	EIAQDFkT	[a8]2+	457.2804	-0.503	-1099.981615
457.4198	5	EIAQDFkT	[a8]2+	457.2804	0.139	303.9710627
296.2281	15	EIAQDFkT	[a8]3+	295.8577	0.37	1250.601218
399.5728	12	EIAQDFkTDL	[b10]3+	399.8896	-0.317	-792.7187507
243.2133	3	EI	[b2]1+	243.1339	0.079	324.9237982
314.5323	2	EIA	[b3]1+	314.1711	0.361	1149.055586
557.1471	15	EIAQD	[b5]1+	557.2566	-0.109	-195.6011035
542.8486	4	EIAQDFkTD	[b9]2+	542.7888	0.06	110.5402404
686.3391	4	EIAQDFkTDLR	[p0]2+	686.3866	-0.048	-69.93143209
458.393	7	EIAQDFkTDLR	[p0]3+	457.9268	0.466	1017.629803
457.4198	5	EIAQDFkTDLR	[p0]3+	457.9268	-0.507	-1107.163756
288.2638	27	LR	[y2]1+	288.203	0.061	211.6563525
504.3421	12	TDLR	[y4]1+	504.2776	0.064	126.9142134
334.8869	11	kTDLR	[y5]2+	334.7278	0.159	475.0128739
408.4251	78	FkTDLR	[y6]2+	408.262	0.163	399.2534402
465.9628	97	DFkTDLR	[y7]2+	465.7755	0.187	401.4810141
529.9102	80	QDFkTDLR	[y8]2+	529.8047	0.105	198.1862223
353.8254	10	QDFkTDLR	[y8]3+	353.5389	0.287	811.7918106
565.5103	100	AQDFkTDLR	[y9]2+	565.3233	0.187	330.7841725
377.3708	84	AQDFkTDLR	[y9]3+	377.218	0.153	405.6010483

Supplementary Table 4: The observed (expt_mz) and corresponding theoretical Mz (theory_mz) for H3K79 with di-methyl heavy labelling ($2[^{13}CD_3] - H_2$) (see Figure 3c in the main manuscript). The difference in Da (dDa) and PPM (dPPM) is provided. Fragmentation data was acquired in a linear ion trap, and database-searched using Mascot with 0.6Da tolerance for fragments and 10ppm for parent ion mass. The table was generated directly from the mass spectrometry Raw files using an in-house script in R. The fragment-type is given (ms2Type) within [ ] with the theoretical charge after the brackets; a=a-ion series, b=b-ion series, p=parent ion, y=y-ion series. Intensity values are normalised by the most intense ion (base-peak normalization).

expt_mz	expt_intensity	ms2seq	ms2type	theory_mz	dDa	dPPM
628.4249	16	QSkQR	[a5]1+	628.3889	0.036	57.28936
129.086	33	Q	[b1]1+	129.0659	0.02	154.9597
216.17	13	QS	[b2]1+	216.0979	0.072	333.1824
372.1617	10	QSk	[b3]1+	372.2242	-0.062	-166.566
186.116	14	QSk	[b3]2+	186.6157	-0.5	-2679.3
500.4296	7	QSkQ	[b4]1+	500.2827	0.147	293.8339
656.4434	60	QSkQR	[b5]1+	656.3838	0.06	91.40993
262.3094	4	QSkQRK	[n0]3+	262.8284	-0.519	-1974.67
402.2995	8	QSkQRK	[p0]2+	401.7483	0.551	1371.505
262.3094	4	QSkQRK	[w0]3+	262.1644	0.145	553.088
147.0805	27	K	[y1]1+	147.1128	-0.032	-217.52
303.6698	5	RK	[y2]1+	303.2139	0.456	1503.889
216.17	13	QRK	[y3]2+	216.1399	0.03	138.799
294.2367	7	kQRK	[y4]2+	294.203	0.034	115.5665
674.4624	30	SkQRK	[y5]1+	674.4308	0.032	47.44742

Supplementary Table 5: The observed (expt_mz) and corresponding theoretical Mz (theory_mz) for H2BWT K60 dimethyl (2[CH_2]) (see Supplementary Figure 6). The difference in Da (dDa) and PPM (dPPM) is provided. Fragmentation data was acquired in a linear ion trap, and database-searched using Mascot with 0.6Da tolerance for fragments and 10ppm for parent ion mass. The table was generated directly from the mass spectrometry Raw files using an in-house script in R. The fragment-type is given (ms2Type) within [ ] with the theoretical charge after the brackets; a=a-ion series, b=b-ion series, p=parent ion, n=parent ion – NH2; w=parent ion – H_2O, w= and y=y-ion series. Intensity values are normalised by the most intense ion (base-peak normalization).

MZ 2[CH3]	Intensity 2[CH3]	MZ CH3.13CD3	Intensity CH3.13CD3	Difference	Type
674.46240	30.45253	678.39325	43.60366	3.93085	Predicted and matched
656.44336	60.18810	660.39600	95.28951	3.95264	Predicted and matched
628.42493	16.34094	632.42810	49.23981	4.00317	Predicted and matched
509.33353	45.14771	513.33820	100.00000	4.00467	Predicted and matched
370.22385	66.79230	374.27512	9.07560	4.05127	Mass-shift only
404.31091	7.37376	408.30508	13.17768	3.99417	Mass-shift only
509.33353	45.14771	513.33820	100.00000	4.00467	Mass-shift only
528.47980	13.95117	532.67688	5.13591	4.19708	Mass-shift only
510.32645	11.85727	514.31012	26.11783	3.98367	Mass-shift only
542.35327	9.95923	546.54492	3.91771	4.19165	Mass-shift only
656.44336	60.18810	660.39600	95.28951	3.95264	Mass-shift only
674.46240	30.45253	678.39325	43.60366	3.93085	Mass-shift only
657.41400	21.38552	661.40961	44.60046	3.99561	Mass-shift only
638.41571	20.16889	642.49762	12.24323	4.08191	Mass-shift only
628.42493	16.34094	632.42810	49.23981	4.00317	Mass-shift only
639.38593	12.95792	643.40546	14.95228	4.01953	Mass-shift only
675.59021	12.72790	679.39319	12.52859	3.80298	Mass-shift only
727.46704	18.76309	731.41602	0.76840	3.94897	Mass-shift only
743.54297	4.40374	747.39801	7.11719	3.85504	Mass-shift only
744.49689	1.74850	748.07941	1.30937	3.58252	Mass-shift only
725.47662	1.21169	729.48016	1.65852	4.00354	Mass-shift only
726.68384	1.09450	730.63763	1.67548	3.95380	Mass-shift only
812.59839	0.55349	816.36829	0.11076	3.76990	Mass-shift only
811.94586	0.41655	816.36829	0.11076	4.42242	Mass-shift only
181.13327	3.68568	183.24612	4.25251	2.11285	Mass-shift only
293.19357	16.06514	295.33334	3.86761	2.13977	Mass-shift only
370.22385	66.79230	372.59473	6.76217	2.37088	Mass-shift only
371.00995	37.14953	373.52548	9.57058	2.51553	Mass-shift only
372.16174	10.24063	374.27512	9.07560	2.11337	Mass-shift only
375.39557	8.42220	377.39316	12.22400	1.99759	Mass-shift only
675.59021	12.72790	677.46765	7.57490	1.87744	Mass-shift only
727.46704	18.76309	729.48016	1.65852	2.01312	Mass-shift only
728.53741	8.97361	730.63763	1.67548	2.10022	Mass-shift only
729.45813	2.90114	731.41602	0.76840	1.95789	Mass-shift only
814.34863	0.16839	816.36829	0.11076	2.01965	Mass-shift only

Supplementary Table 6: Masses from fragmentation spectra for H2BWT-K60 2[CH_3] labelling and H2BWT-K60 $CH_3.^{13}CH_3$ that differ due to the incorporation of a heavy isotopes (4.02218 Da) were identified using HiLight-PTM (see Supplementary Figure 6). Peaks that match exactly will not carry the di-methyl label, whereas their counter-fragment will. Therefore, if we observe exact matched peaks between the two spectra, we can predict the presence of the labelled-equivalent, these are indicated as “Prediction and matched”. “Mass-shift only” means there was no unlabeled counter-fragment identified. The intensity of the matches ions were normalised by the most intense peak in their respective spectra (base-peak normalised) and correlate with r=0.8 (P<0.001, Pearson’s correlation coefficient)

Supplementary Figures

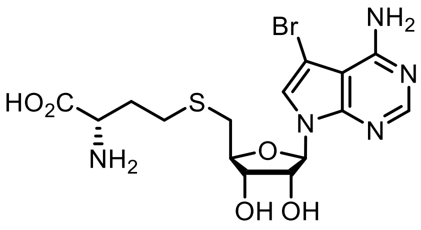

Supplementary Figure 1: Structure of the selective DOT1L inhibitor, bromo-deaza-SAH[10].

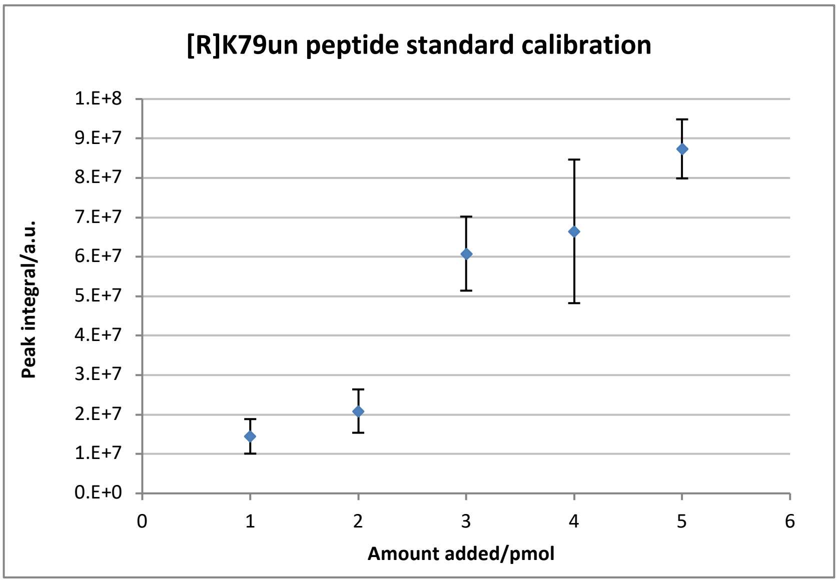

Supplementary Figure 2: Plot of the MS peak integrals recorded for the unmodified K79-containing peptide when added to the unreacted nucleosome sample. a.u. = arbitrary units, [R] = heavy arginine residue at C-terminus. Error = s.d. between three replicates.

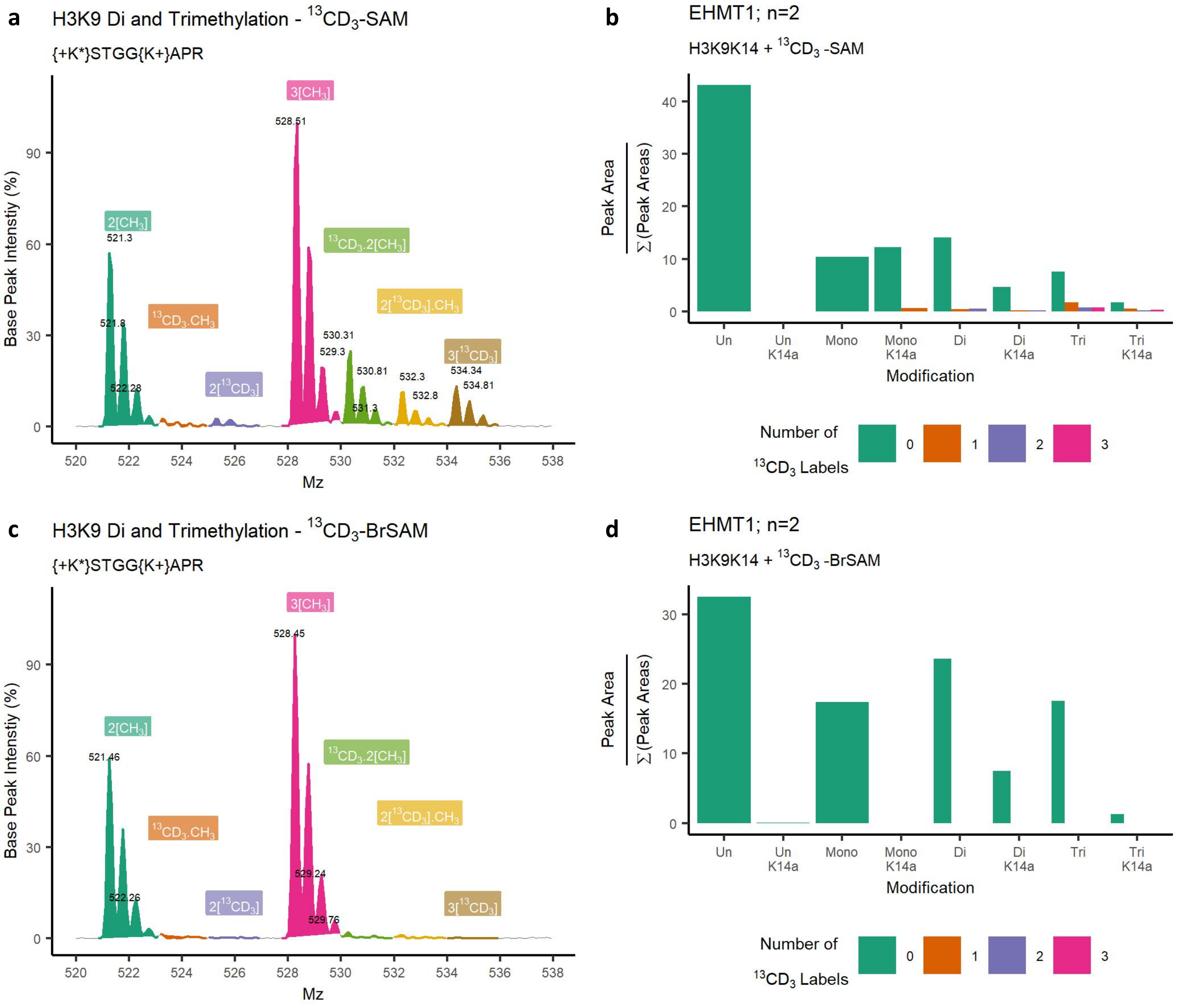

Supplementary Figure 3: Methylation of nucleosomes in the presence of EHMT1/G9A. (a) representative mass spectra of H3K9K14 mono and di methylation with incorporation of $^{13}CD_3$ when reacted with $^{13}CD_3$-SAM, (b) relative proportion of labelled proteoforms. When reacted with $^{13}CD_3$-BrSAM, no heavy labels are observed in either (c) the MS or (d) chromatographic peak integrals.

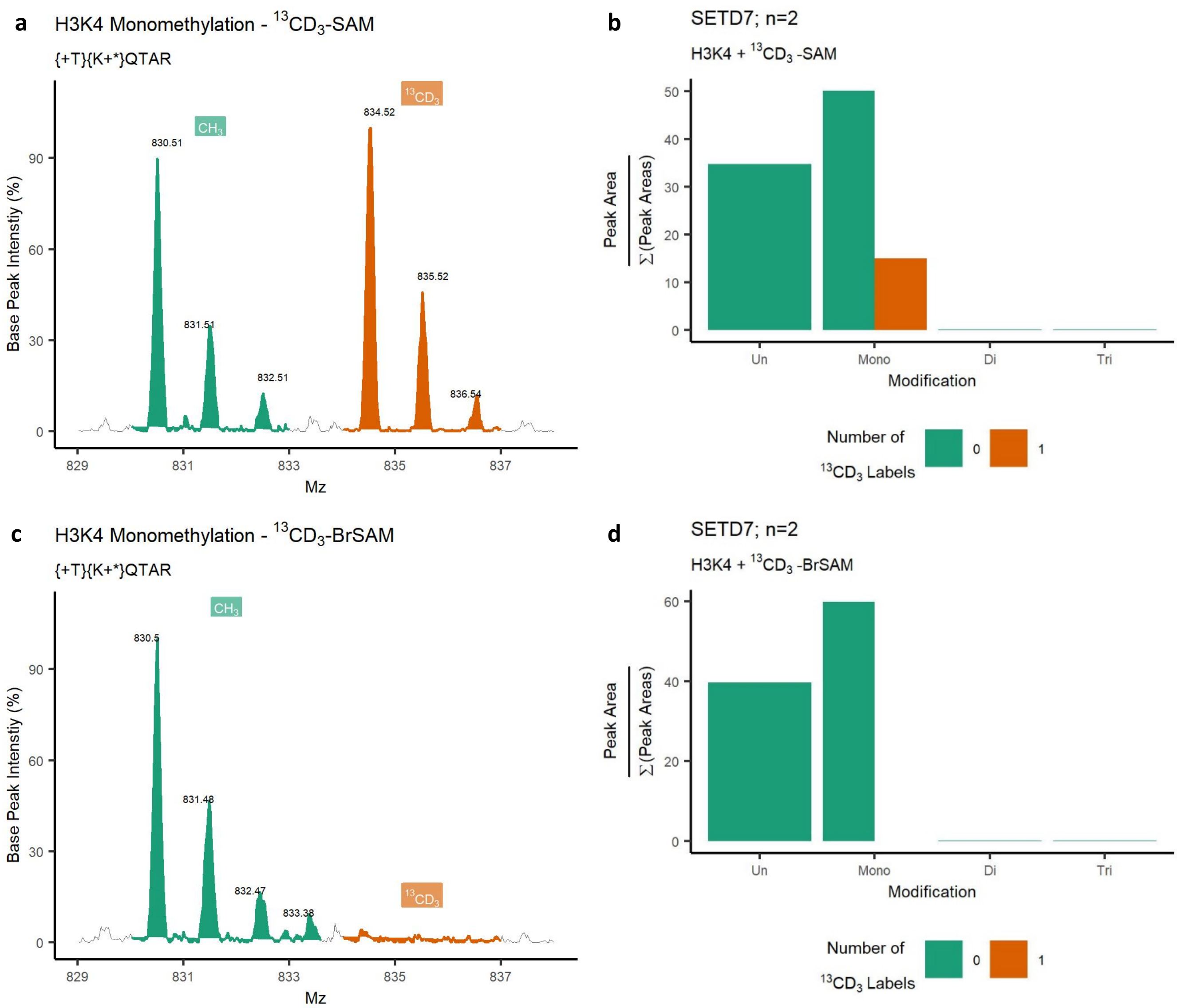

Supplementary Figure 4: Methylation of nucleosomes in the presence of SETD7. (a) representative mass spectra of H3K4 mono and di methylation with incorporation of $^{13}CD_3$ when reacted with $^{13}CD_3$-SAM, (b) relative proportion of labelled proteoforms. When reacted with $^{13}CD_3$-BrSAM, no heavy labels are observed in either (c) the MS or (d) chromatographic peak integrals.

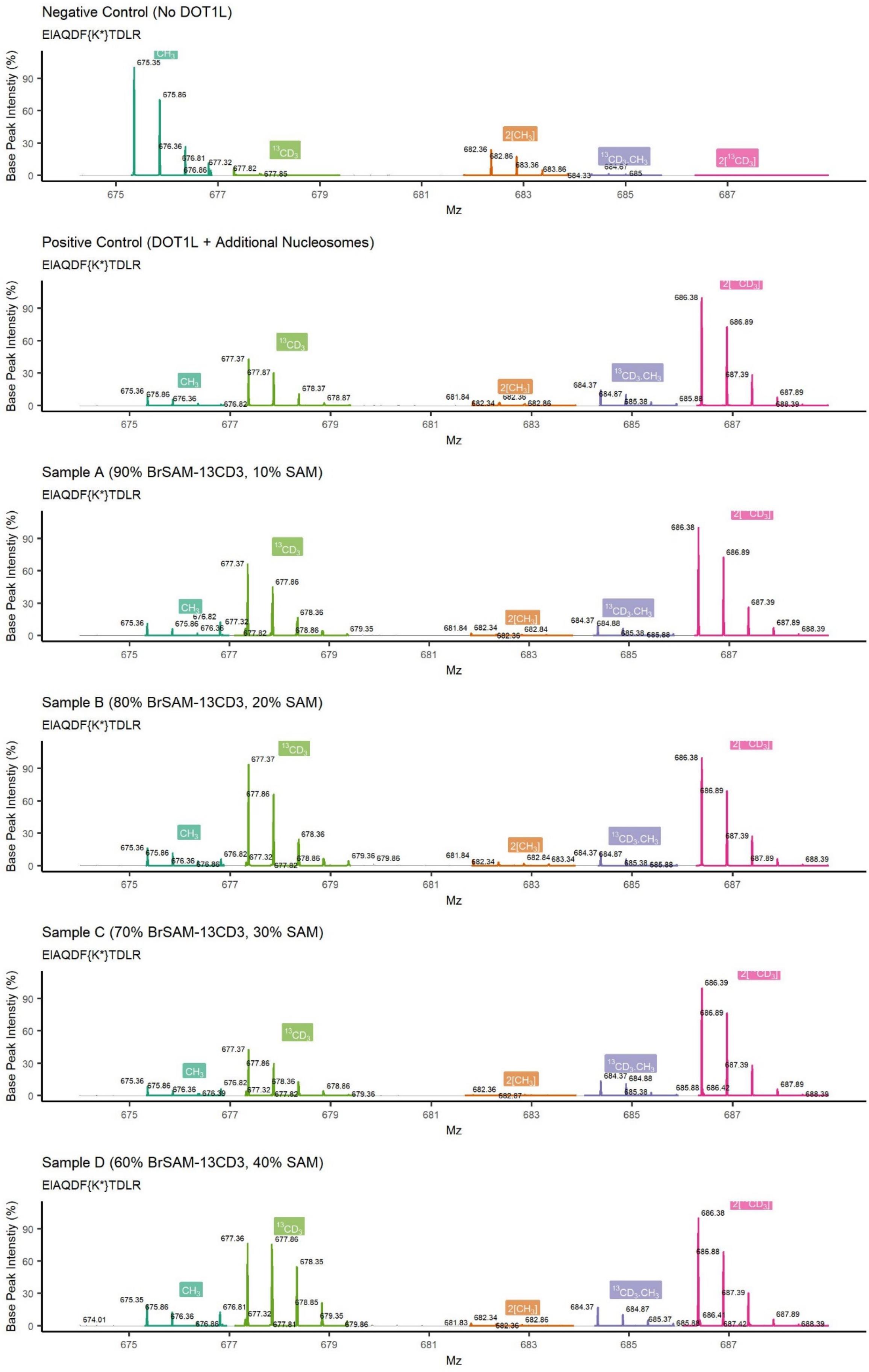
Negative Control (No DOT1L)
EIAQDF{K*}TDLR
Positive Control (DOT1L + Additional Nucleosomes)
EIAQDF{K*}TDLR
Sample A (90% BrSAM-13CD3, 10% SAM)
EIAQDF{K*}TDLR
Sample B (80% BrSAM-13CD3, 20% SAM)
EIAQDF{K*}TDLR
Sample C (70% BrSAM-13CD3, 30% SAM)
EIAQDF{K*}TDLR
Sample D (60% BrSAM-13CD3, 40% SAM)
EIAQDF{K*}TDLR
Base Peak Intensity (%)
Mz
CH3
13CD3
2[CH3]
13CD3.CH3
2[13CD3]

Supplementary Figure 5: MS1 scans showing the putative presence of H3K79 mono-methyl and di-methyl peaks. The positive control, and samples A-D, show the incorporation of heavy labelling, whereby peaks within the regions labelled $^{13}CD_3$ (light-green), $^{13}CD_3.CH_3$ (purple) and $2[^{13}CD_3]$ (pink), indicating a reaction with BrSAM-$^{13}CD_3$. In the negative control (no DOT1L), only endogenous methylation (no heavy label) can be seen in the regions labelled CH_3 (dark green) and $2[CH_3]$ (orange).

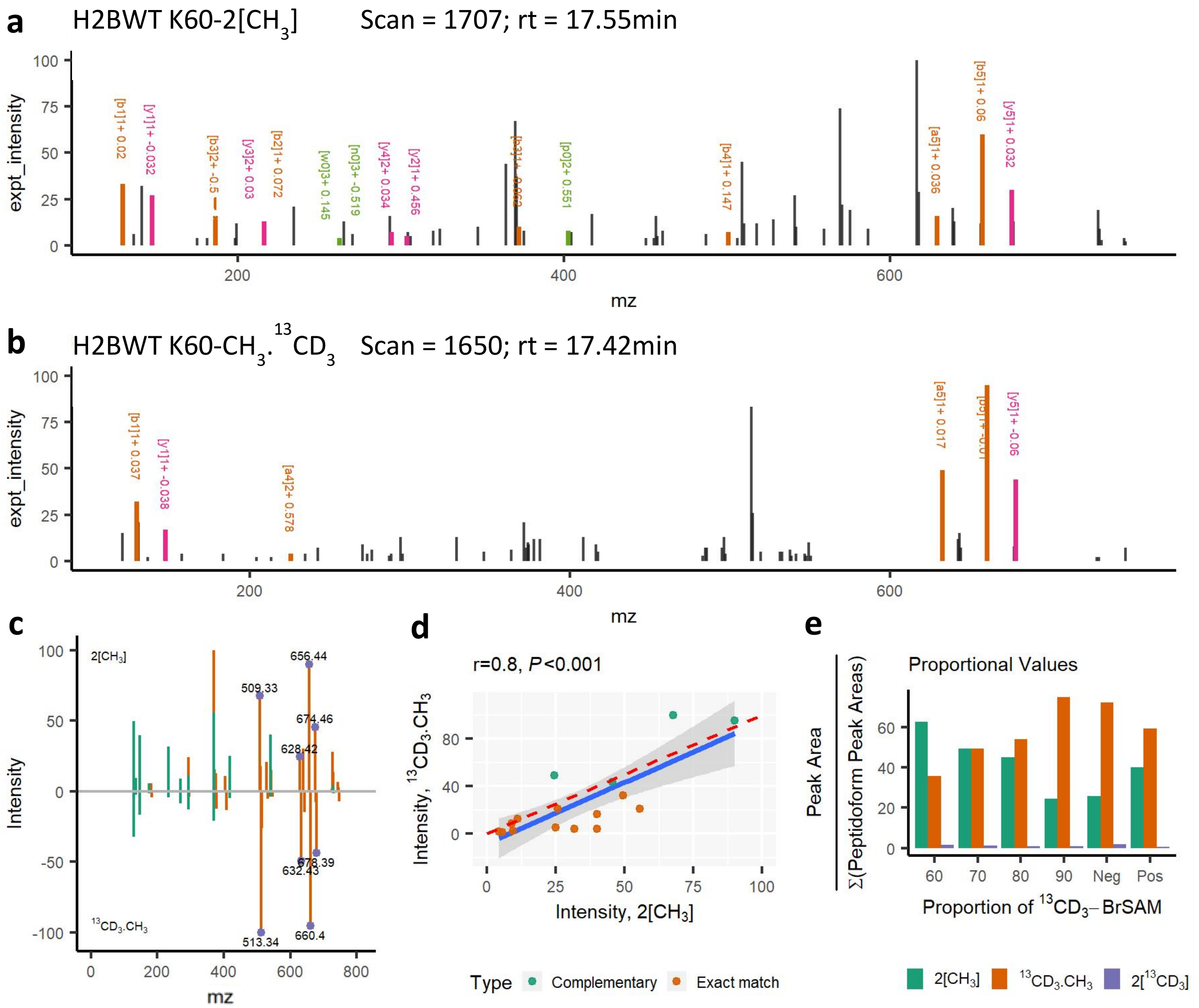

Supplementary Figure 6 (a) K60 di-methylation (2[CH3]) of H2BWT was identified following database searching, and (b) an MS2 spectra from a precursor corresponding to a +4 shift was identified from (c) an in-house script that looks for peaks that are common (green) or complementary (orange with blue dot/mass annotation). (d) The Pearson's product moment correlation coefficient between matching (green) and complementary (orange) fragment ions was calculated. A linear regression (blue line) with 95% confidence intervals (shading), and the line y=x is given for comparison (red dashed line). (e) The proportion of peptides with light (green) or heavy (orange) methylation was determined for each experimental condition (60% - 90% $^{13}CD_3$-BrSAM, positive control (supplementation with nucleosomes) negative control (without exogenous DOT1L)). In mass spectra (a & b), orange = a/b-ion series, pink = y-ion series, green = parent ion. The mass difference from the theoretical mass is given by each peak in Da.

Chemical synthesis

General methods

7-bromo-6-chloro-7-deazapurine **1** was purchased from Fluorochem, all other chemicals were purchased from Sigma Aldrich. SAH, SAM and related compounds were stored below -20 °C. All solvents used were dry unless otherwise indicated. Reactions were performed on an IKA magnetic hotplate stirrer unless otherwise stated. Microwave irradiation was carried out using a Discoverer SP system (CEM Technology). Reactions were monitored using TLC plates purchased from Merck (60 F_{254}, 0.25 mm). Column chromatography was carried out using either silica gel (40-63 µm 60 Å, Merck) for normal phase separation or Amberlite XAD-4 resin (Sigma Aldrich) for reverse phase separation. Isolated compounds were dried using a Büchi R-114 rotary evaporator. HPLC fractions were dried using a speedvac concentrator (Thermo Scientific Savant). Compound purity was assessed by TLC, NMR and MS.^{1}H and ^{13}C NMR for the majority of compounds were recorded using a Bruker Advance 400 Spectrometer. The samples were prepared in an appropriate deuterated solvent (purchased from Cambridge Isotope Laboratories or Sigma Aldrich) and the internal reference was Me_4Si. For compound **7**, 200 µL of aqueous solution of $^{13}CD_3$-BrSAM were mixed with 20 µL of potassium phosphate buffer (1.5 M in D_2O at pH 7.4, containing NaN_3 2 mM and 5.8 mM TSP as a reference). The solution was introduced in a 3 mm NMR tube and analysed in a Bruker Avance III 600 equipped with a BBI probe working at room temperature. Mass spectra (electrospray ionisation or chemical ionisation: ESI, CI) were recorded by the Mass Spectrometry Service of Imperial College London, Department of Chemistry using a Micromass Autospec Premier and Micromass LCT Premier spectrometer. Alternatively, LC-MS data was obtained using an LTQ Velos Pro linear ion trap LC-MS system (Thermo Scientific). Electrospray ionisation (ESI) was the primary ionisation method over a mass range of 200−2000 m/z and a secondary CID fragmentation was applied with a collision energy of 35%. Identification of the ion of interest was conducted through product fragment comparisons with theoretical fragmentation of the desired chemical structure. The samples were eluted over a linear gradient of 100% solvent A (99.9% H_2O, 0.1% FA) to 5% A and 95% B (95% MeCN, 4.9% H_2O, 0.1% FA) over 10 minutes, at which point the gradient remained isocratic for 2 minutes.

For HPLC purifications, crude products were fractioned by UV peak detection (215 nm) using a Dionex Ultimate 3000 UHPLC system (Thermo Scientific) using a Supelco Ascentis Express C18 column (2.7 µm particle size, 150 mm x 4.6 mm, Sigma Aldrich). The LC conditions comprised of a flow rate of 0.5 mL/min and a multi-step gradient starting at 0.5% B (95% MeCN, 4.8% H_2O and 0.2% TFA or 0.2% FA) and 99.5% A (5% MeCN, 94.8% H_2O and 0.2% TFA or 0.2% FA) for two minutes, then linearly increased to 20% B over 15 minutes, followed by linear increase to 95% B over a further 7 minutes. All solvents used were HPLC grade (Sigma Aldrich or Thermo Fisher). To identify the purified peak of interest, fractions were further analyzed using LC-MS as described above.

Synthetic route

The synthesis of bromo-deaza-SAH **5** was adapted from the original route published by Yu *et al*[10]. A procedure for the methylation of SAH/BrSAH was taken from the method reported by Kuethe[11].

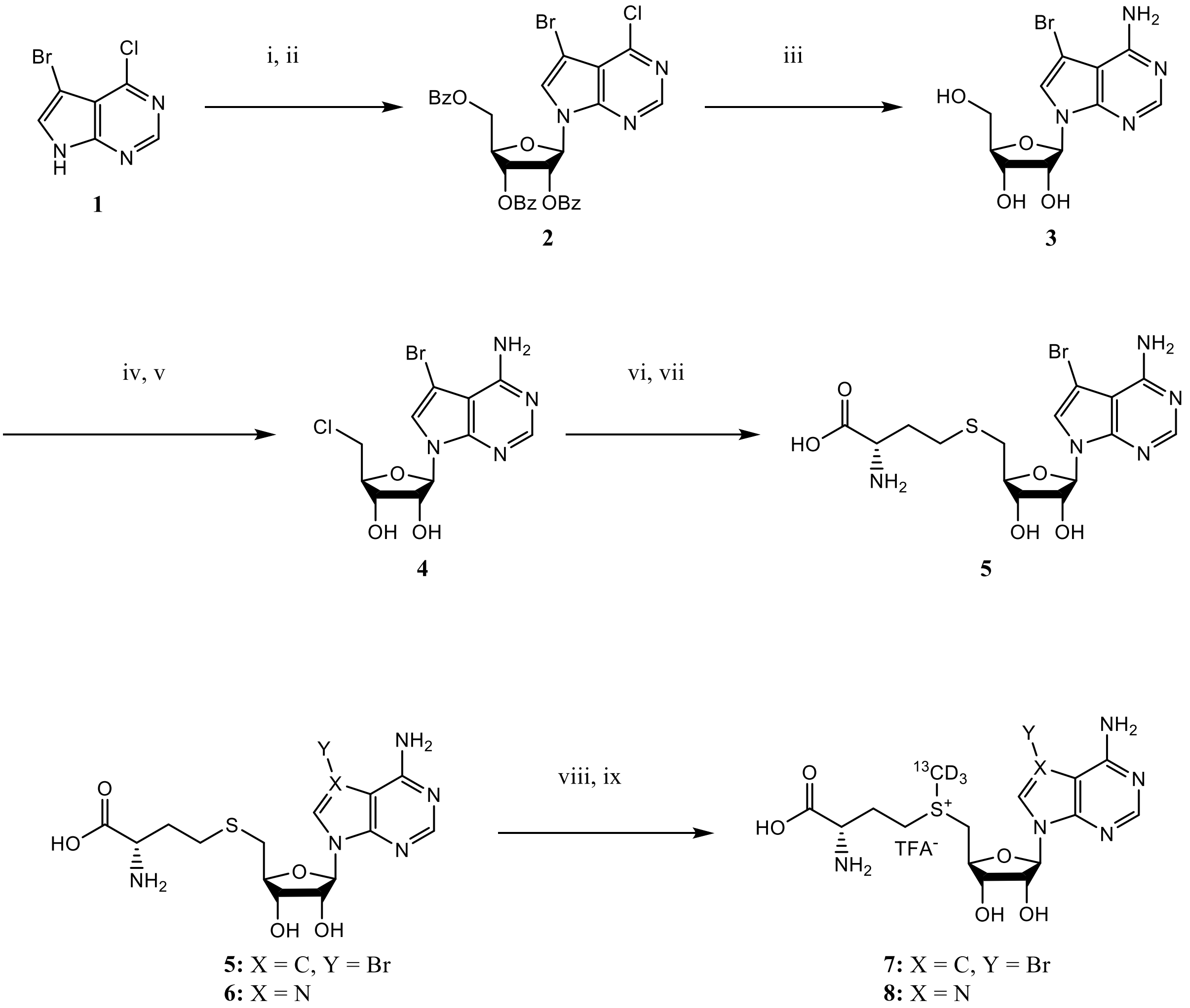

Scheme: (i) BSA, MeCN, RT, 30 min; (ii) 1-*O*-acetyl-2,3,5-tri-*O*-benzoyl-ß-D-ribofuranose, TMSOTf, 80 °C, 1 hour; (iii) NH_3/MeOH, 120 °C, 1 hour under microwave irradiation; (iv) $SOCl_2$, pyridine, MeCN, RT, 18 hours; (v) NH_4OH, MeOH/H_2O (5:1), RT, 30 min; (vi) *L*-homocysteine thiolactone hydrochloride, NaOH, H_2O, RT, 10 min; (vii) **4**, KI, 80 °C, 16 hours; (viii) $^{13}CD_3I$, AgOTf, CCl_4, RT, 10 min; (ix) **5** or **6**, HCOOH, RT, 18 hours.

Synthetic procedures

(2*R*,3*R*,4*S*,5*R*)-2-(benzoyloxymethyl)-5-(5-bromo-4-chloro-*7H*-pyrrolo[2,3-*d*]pyrimidin-7-yl)tetrahydrofuran-3,4-diyl dibenzoate 2

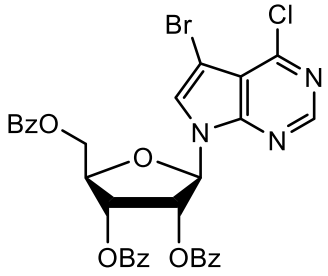

To a suspension of 7-bromo-6-chloro-7-deazapurine **1** (1.5 g, 6.45 mmol) in MeCN (60 mL) was added *bis*-trimethylsilylacetamide (1.89 mL, 7.74 mmol). The mixture was stirred at RT for 30 minutes. 1-*O*-acetyl-2,3,5-tri-*O*-benzoyl-ß-D-ribofuranose (3.90 g, 7.74 mmol) was added, followed by TMSOTf (1.4 mL, 7.74 mmol). The reaction mixture was heated to 80 °C and stirred for 1 hour. The resultant orange-brown solution was left to cool and concentrated using rotary evaporation. After dilution with EtOAc (30 mL), the mixture was partitioned with sat. $NaHCO_3$ (20 mL) and extracted with EtOAc (3 x 20 mL). The combined organics were dried over Na_2SO_4 and evaporated to give a crude orange foam. Purification through a silica gel column with pentane/EtOAc (4:1) gave the desired product **2** as a white foam (3.40 g, 5.03 mmol).

Yield 78% ; δ_{1H}/ppm ($CDCl_3$, 400 MHz, Me_4Si) .8.61 (s, 1H), 8.19 – 8.04 (m, 3H), 8.07 – 7.88 (m, 5H), 7.69 – 7.39 (m, 11H), 7.39 (d, *J* = 7.8 Hz, 2H), 7.29 (s, 1H), 6.71 (d, *J* = 5.3 Hz, 1H), 6.26 – 6.09 (m, 2H), 4.93 (dd, *J* = 12.3, 3.1 Hz, 1H), 4.83 (q, *J* = 3.5 Hz, 1H), 4.71 (dd, *J* = 12.3, 3.6 Hz, 1H), 4.15 (q, *J* = 7.2 Hz, 1H), 2.07 (s, 1H), 1.61 (s, 3H), 1.29 (t, *J* = 7.1 Hz, 2H) ; δ_{13C}/ppm ($CDCl_3$, 100 MHz, Me_4Si) 166.1, 165.4, 165.1, 152.8, 151.6, 150.8, 133.8, 133.6, 129.9, 129.7, 129.3, 128.8, 128.7, 128.6, 128.5, 128.4, 126.5, 116.0, 90.2, 86.8, 80.7, 74.1, 71.4, 63.5, 14.2 ; MS (ESI) *m/z* 676.0, 678.0 $[M+H]^+$, 679.1 and 680.1.

(2*R*,3*R*,4*S*,5*R*)-2-(4-amino-5-bromo-*7H*-pyrrolo[2,3-*d*]pyrimidin-7-yl)-5-(hydroxymethyl)tetrahydrofuran-3,4-diol 3

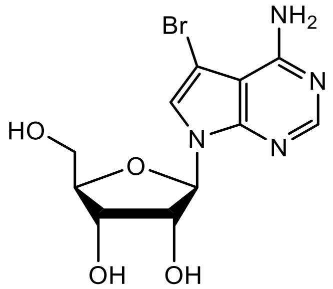

A suspension of **2** (500 mg, 0.74 mmol) in *ca.* 7 M NH_3/MeOH solution (20 mL) was prepared in a microwave vial and stirred at RT for 10 minutes. It was then transferred to a microwave reactor, in which it was heated to 120 °C with stirring for 1 hour. The solvent was removed by rotary evaporation to give a crude dark orange residue, which was purified through a silica gel column with a stepwise gradient of: i) CH_2Cl_2, ii) CH_2Cl_2/MeOH [7 M NH_3] (93:7) and iii) CH_2Cl_2/MeOH [7 M NH_3] (9:1) to yield the desired product **3** as an orange amorphous solid (115 mg, 0.33 mmol).

Yield 45% ; δ_{1H}/ppm (DMSO-d_6, 400 MHz, Me_4Si) 8.11 (s, 1H), 7.67 (s, 1H), 6.86 (br s, 2H), 6.05 (d, *J* = 6.2 Hz, 1H), 5.36 (d, *J* = 6.4 Hz, 1H), 5.17 (m, 2H), 4.40 – 4.32 (m, 1H), 4.15 – 4.05 (m, 1H), 3.89 (m, 1H),

3.67 – 3.49 (m, 2H) ; δ_{13C}/ppm (DMSO-*d*$_6$, 100 MHz, Me_4Si) 157.4, 152.9, 150.1, 128.8, 101.5, 87.3, 87.2, 85.7, 74.4, 71.0, 62.0 ; MS (ESI) *m/z* 345.0, 346.0 $[M+H]^+$, 347.0 and 348.0.

(2*R*,3*R*,4*S*,5*R*)-2-(4-amino-5-bromo-*7H*-pyrrolo[2,3-*d*]pyrimidin-7-yl)-5-(chloromethyl)tetrahydrofuran-3,4-diol 4

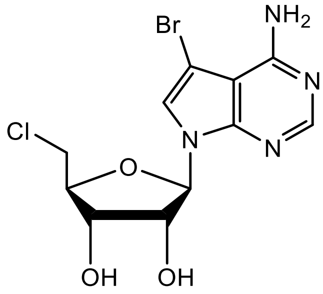

To a suspension of **3** (150 mg, 0.43 mmol) in MeCN (2 mL) at 0 °C was added pyridine (120 µL, 0.87 mmol). After 5 minutes, $SOCl_2$ (126 µL, 1.74 mmol) was slowly added, resulting in a yellow precipitate. The mixture was left to stir at 0 °C for 30 minutes and then allowed to warm to RT and stir for a further 16 hours. The resultant off-white precipitate was filtered, washed with MeCN and dried *in vacuo*. The off-white solid was dissolved in a mixture of MeOH/H_2O (5:1 *v/v*, 1.5 mL) at RT. To this was added conc. NH_4OH solution (0.21 mL, 5.38 mmol) and the reaction was stirred for 30 minutes. The solvent was then evaporated and the crude orange residue was crystallised in H_2O, filtered and dried *in vacuo* to give **4** as an off-white solid (134 mg, 0.37 mmol).

Yield 87% ; δ_{1H}/ppm (DMSO-*d*$_6$, 400 MHz, Me_4Si) 8.13 (s, 1H), 7.65 (s, 1H), 6.85 (br s, 2H), 6.12 (d, *J* = 6.2 Hz, 1H), 5.52 (d, *J* = 6.3 Hz, 1H), 5.43 (d, *J* = 5.0 Hz, 1H), 4.48 (dd, *J* = 11.6, 4.9 Hz, 1H), 4.13-4.00 (m, 2H), 3.94-3.79 (m, 2H) ; δ_{13C}/ppm (DMSO-*d*$_6$, 100 MHz, Me_4Si) 157.4, 153.1, 150.5, 121.9, 101.5, 87.9, 86.4, 83.7, 73.5, 71.6, 45.4 ; MS (ESI) *m/z* 363.0, 365.0 $[M+H]^+$ and 367.0.

(*S*)-2-amino-4-(((2*S*,3*S*,4*R*,5*R*)-5-(4-amino-5-bromo-*7H*-pyrrolo[2,3-*d*]pyrimidin-7-yl)-3,4-dihydroxytetrahydrofuran-2-yl)methylthio)butanoic acid (bromo-deaza-SAH) 5

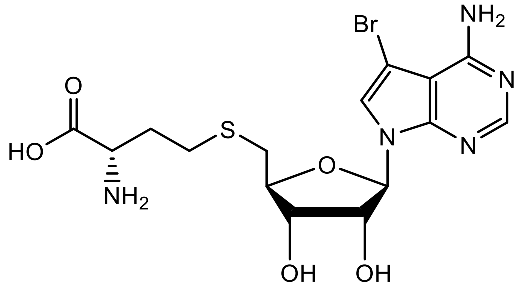

A mixture of *L*-homocysteine thiolactone hydrochloride (250 mg, 1.63 mmol) and NaOH (195 mg, 4.85 mmol) was stirred in water (7.5 mL) at RT under N_2 for ten minutes. To this was added **4** (295 mg, 0.81 mmol) and potassium iodide (40 mg, 0.24 mmol) and the mixture was heated to 100 °C for 16 hours. The solvent was then removed *in vacuo* and replaced with 1 mL of water. The solution was cooled in an ice bath and adjusted to pH 7-8 using dropwise addition of 2 M HCl. An off-white precipitate formed and was left to stand for fifteen minutes. The supernatant liquid was removed using a syringe. This washing method was repeated three times. The off-white solid was dried *in vacuo* (165 mg, 0.36 mmol).

Yield 44% ; δ_{1H}/ppm (DMSO-*d*$_6$, 400 MHz, Me_4Si) 8.12 (s, 1H), 7.66 (s, 1H), 6.82 (s, 2H), 6.08 (d, *J* = 6.2 Hz, 1H), 4.46 (t, *J* = 5.7 Hz, 1H), 4.04 (dd, *J* = 5.2, 3.3 Hz, 1H), 3.96 (m, 1H), 3.25 (m, 1H), 2.88 (dd, *J* =

13.6, 6.5 Hz, 1H), 2.75 (dd, J = 13.6, 6.5 Hz, 1H), 2.63 (t, J = 7.7 Hz, 2H), 1.99 (m, 1H), 1.81 (m, 1H) ; δ_{13C}/ppm (DMSO-d_6, 100 MHz, Me_4Si) 157.5, 153.1, 150.5, 122.1, 101.4, 87.7, 86.9, 83.5, 73.5, 72.9, 53.5, 34.4, 31.8, 28.5 ; LC-MS (ESI) *m/z* 462.1, 464.1 $[M+H]^+$, 465.2 and 466.1.

((*S*)-3-amino-3-carboxypropyl)(((2*S*,3*S*,4*R*,5*R*)-5-(4-amino-5-bromo-*7H*-pyrrolo[2,3-*d*]pyrimidin-7-yl)-3,4-dihydroxytetrahydrofuran-2-yl)methyl)(methyl-13C-*d3*)sulfonium 2,2,2-trifluoroacetate ($^{13}CD_3$-BrSAM) 7

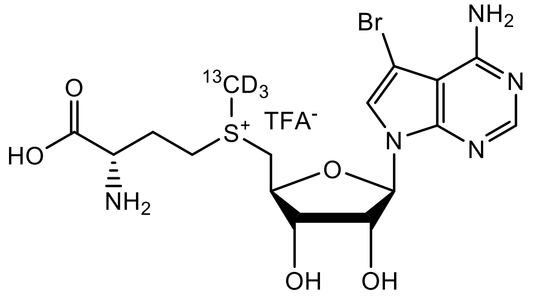

5 (4 mg, 0.0086 mmol) was dissolved in neat HCOOH (1 mL). A separate suspension of AgOTf (104 mg, 0.41 mmol) and CD_3I (252 µL, 4.1 mmol) in CCl_4 (1 mL) was stirred for 10 minutes at RT under N_2, after which time a bright yellow precipitate had formed. The mixture was filtered over a syringe pad and the filtrate was added to the solution of **5**. High-speed stirring was maintained for 24 hours. Water (1 mL) was added to the reaction mixture, which was subsequently dried *in vacuo*. The product was purified by HPLC under the conditions outlined in the general methods (the product eluted at 5.6 min)to give a clear residue (1.2 mg, 0.0020 mmol).

Yield 25%; δ_{1H}/ppm (1.5 M potassium phosphate buffer in D_2O at pH 7.4, 600 MHz) 8.16 (s, 1H), 7.45 (s, 1H), 6.19 (m, 1H), 4.51-4.44 (m, 1H), 4.33-4.54 (m, 2H), 4.01-3.90 (m, 1H), 3.89-3.70 (m, 2H), 3.63-3.46 (m, 2H), 2.36-2.24 (m, 2H) ; δ_{13C}/ppm* (1.5 M potassium phosphate buffer in D_2O at pH 7.4, 600 MHz, 151 MHz) 155.2, 124.5, 124.2, 91.0, 80.8, 80.4, 75.8, 75.5, 55.8, 47.3, 47.2, 47.1, 46.9, 41.8, 28.4, 26.0 (septet, J_{CD} = 21.1 Hz) ; LC-MS (ESI) m/z 480.0857 $[M]^+$.

Mixture of diastereomers, all observed signals reported. *All signals except $^{13}CD_3$ reported using HSQC.

(2*S*)-2-amino-4-((((2*S*,3*S*,4*R*,5*R*)-5-(6-amino-*9H*-purin-9-yl)-3,4-dihydroxytetrahydrofuran-2-yl)methyl)(methyl-13C-*d3*)-l4-sulfaneyl)butanoic acid, 2,2,2-trifluoroacetate ($^{13}CD_3$-SAM) 8

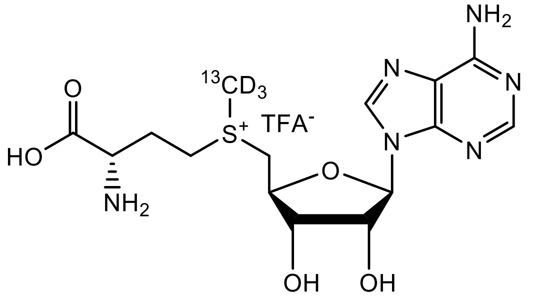

8 was prepared from **6 (SAH)** (10 mg, 0.026 mmol) and $^{13}CD_3I$ using the procedure for compound **7**. The product was purified by HPLC under the conditions outlined in the general methods (the product eluted at 4.0 min) to give a clear residue (4.4 mg, 0.009 mmol).

Yield 33% ; δ_{1H}/ppm (D_2O, 400 MHz, Me_4Si) 8.40 (s, 1H), 8.39 (s, 1H), 6.17 (d, J = 3.9 Hz, 1H), 4.85 (q, J = 4.7 Hz, 1H), 4.66 – 4.48 (m, 2H), 3.98 – 3.83 (m, 3H), 3.68 – 3.38 (m, 2H), 2.33 (m, 2H) ; δ_{13C}/ppm* (D_2O, 100 MHz, Me_4Si) 143.8, 90.0, 78.5, 73.1, 72.7, 51.8, 43.9, 38.5, 24.6, 23.0 (septet, J_{CD} = 22.0 Hz) ; LC-MS (ESI) m/z 403.18 $[M]^+$.

Mixture of diastereomers, all observed signals reported. *All signals except $^{13}CD_3$ reported using HSQC.

Spectra for 7 and 8: Supplementary Figures 7-11

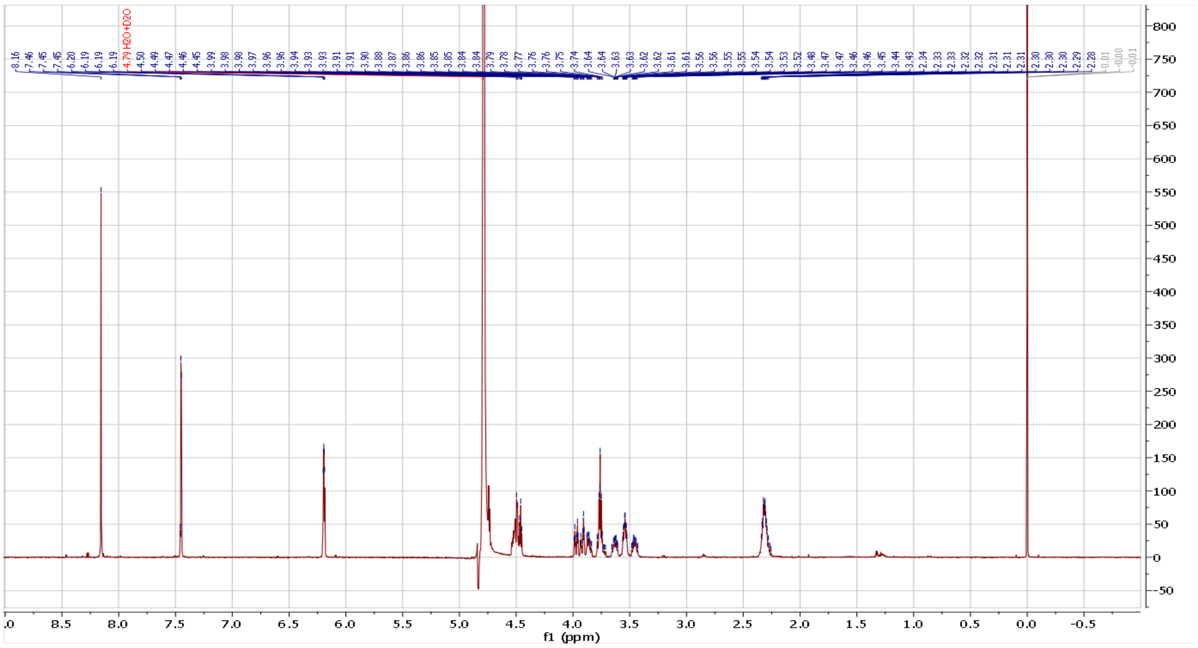

Supplementary Figure 7: ^{1}H NMR spectrum of $^{13}CD_3$-BrSAM, **7**

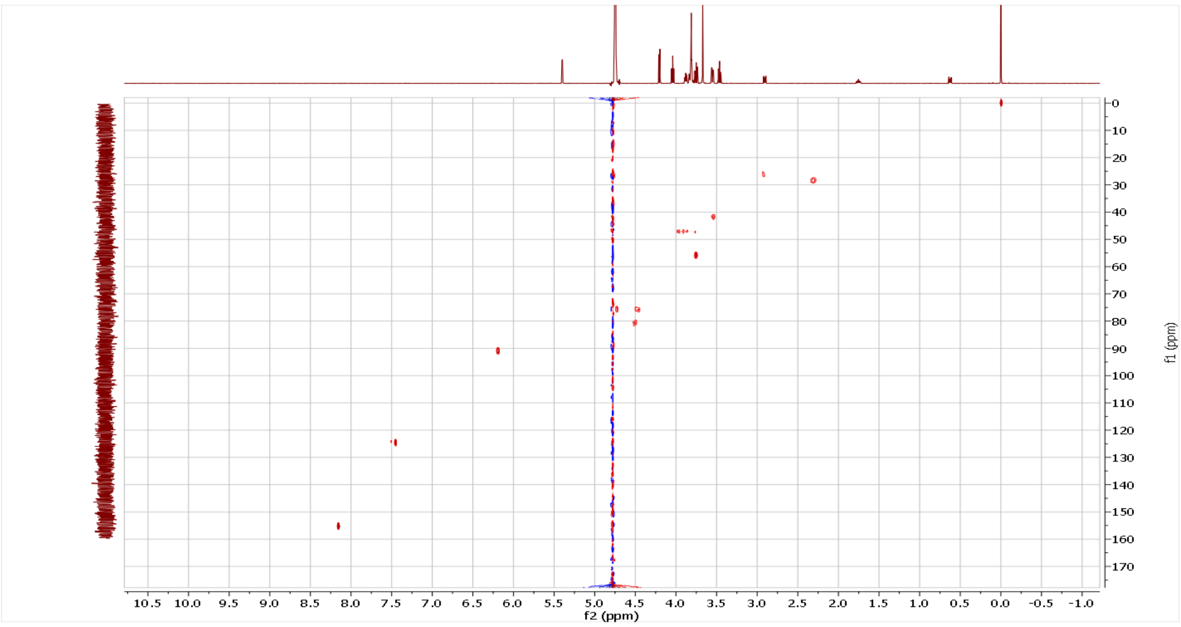

Supplementary Figure 8: HSQC NMR spectrum of $^{13}CD_3$-BrSAM, **7**

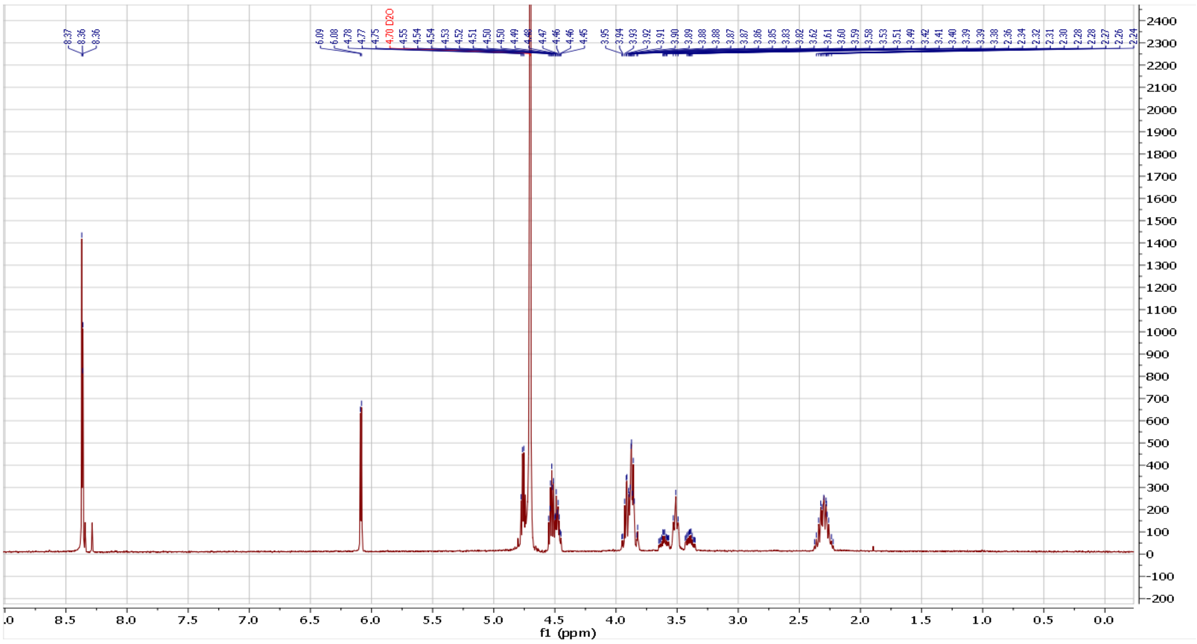

Supplementary Figure 9: ^{1}H NMR spectrum of $^{13}CD_3$-SAM, **8**

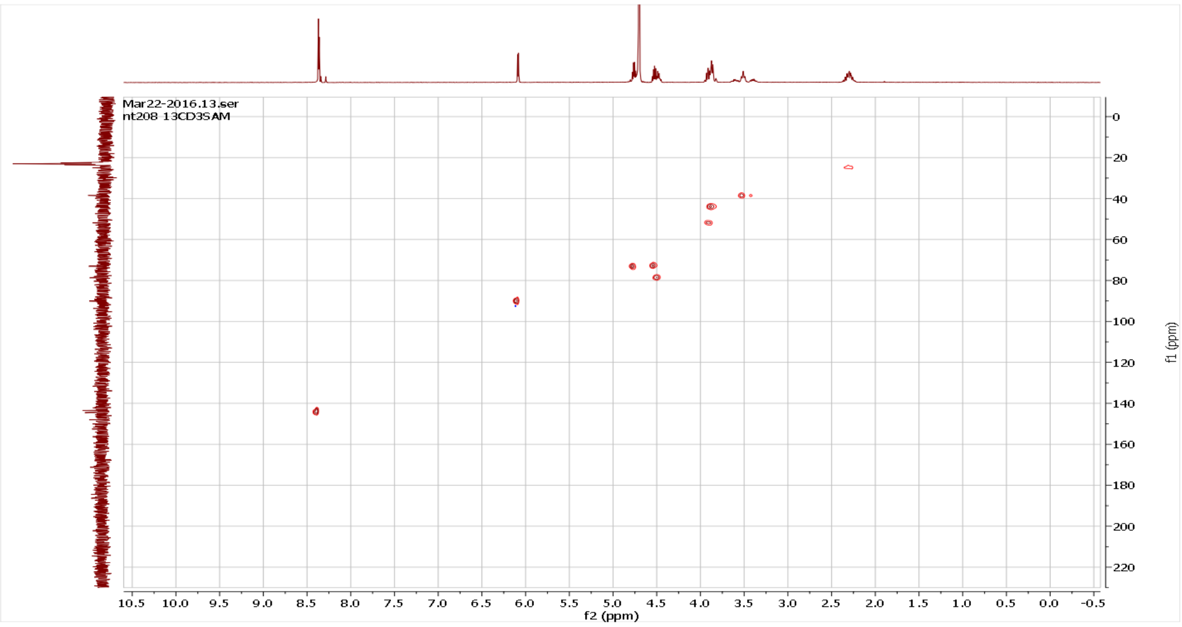

Supplementary Figure 10: HSQC spectrum of $^{13}CD_3$-SAM, **8**

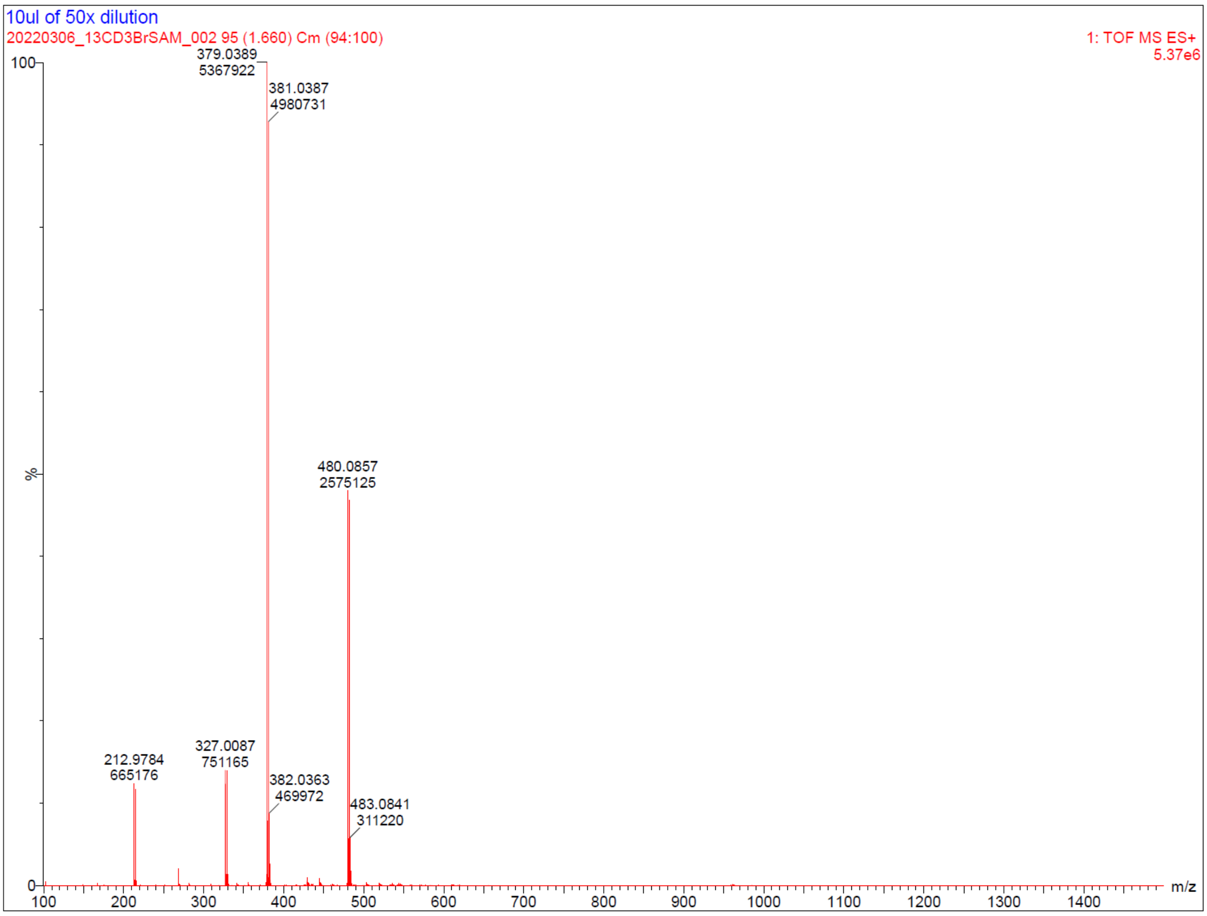

Supplementary Figure 11: HRMS of $^{13}CD_3$-BrSAM, **7**

Supplementary References

1 Nguyen, H. *et al.* LLY-507, a Cell-active, Potent, and Selective Inhibitor of Protein-lysine Methyltransferase SMYD2 *. *Journal of Biological Chemistry* **290**, 13641-13653 (2015). https://doi.org:10.1074/jbc.M114.626861

2 Chambers, M. C. *et al.* A cross-platform toolkit for mass spectrometry and proteomics. *Nature Biotechnology* **30**, 918-920 (2012). https://doi.org:10.1038/nbt.2377

3 Whitwell, H. J. & DiMaggio, P. HiLight-PTM: an online application to aid matching peptide pairs with isotopically labelled PTMs. *Bioinformatics* **36**, 938-939 (2020). https://doi.org:10.1093/bioinformatics/btz654

4 Lin, S. & Garcia, B. A. Examining histone posttranslational modification patterns by high-resolution mass spectrometry. *Methods Enzymol* **512**, 3-28 (2012). https://doi.org:10.1016/b978-0-12-391940-3.00001-9

5 Lin, Q., Jiang, F., Schultz, P. G. & Gray, N. S. Design of allele-specific protein methyltransferase inhibitors. *J Am Chem Soc* **123**, 11608-11613 (2001). https://doi.org:10.1021/ja011423j

6 Li, J., Wei, H. & Zhou, M. M. Structure-guided design of a methyl donor cofactor that controls a viral histone H3 lysine 27 methyltransferase activity. *J Med Chem* **54**, 7734-7738 (2011). https://doi.org:10.1021/jm201000j

7 Islam, K. *et al.* Defining efficient enzyme–cofactor pairs for bioorthogonal profiling of protein methylation. *Proceedings of the National Academy of Sciences*, 201216365 (2013). https://doi.org:10.1073/pnas.1216365110

8 Bothwell, I. R. *et al.* Se-Adenosyl-l-selenomethionine Cofactor Analogue as a Reporter of Protein Methylation. *Journal of the American Chemical Society* **134**, 14905-14912 (2012). https://doi.org:10.1021/ja304782r

9 Sohtome, Y., Shimazu, T., Shinkai, Y. & Sodeoka, M. Propargylic Se-adenosyl-l-selenomethionine: A Chemical Tool for Methylome Analysis. *Accounts of Chemical Research* **54**, 3818-3827 (2021). https://doi.org:10.1021/acs.accounts.1c00395

10 Yu, W. *et al.* Bromo-deaza-SAH: a potent and selective DOT1L inhibitor. *Bioorg Med Chem* **21**, 1787-1794 (2013). https://doi.org:10.1016/j.bmc.2013.01.049

11 Kuethe, J. T. Synthesis of stable isotope-labeled metabolites of asenapine. *Journal of Labelled Compounds and Radiopharmaceuticals* **55**, 180-185 (2012). https://doi.org:https://doi.org/10.1002/jlcr.2924
