# Supplementary material for: Tracking DOT1L methyltransferase activity by stable isotope labelling using a selective synthetic co-factor
Source: Commun Chem. 2024 Jun 27;7:145. doi: 10.1038/s42004-024-01227-x (PMC11211345; doi:10.1038/s42004-024-01227-x)
Supplement: Supplementary file 3 — Reporting Summary [file 42004_2024_1227_MOESM3_ESM.pdf]

nature portfolio

Corresponding author(s): Matthew J. Fuchter

Last updated by author(s): Jun 3, 2024

Reporting Summary

Nature Portfolio wishes to improve the reproducibility of the work that we publish. This form provides structure for consistency and transparency in reporting. For further information on Nature Portfolio policies, see our Editorial Policies and the Editorial Policy Checklist.

Statistics

For all statistical analyses, confirm that the following items are present in the figure legend, table legend, main text, or Methods section.

n/a	Confirmed	
☐	☒	The exact sample size (*n*) for each experimental group/condition, given as a discrete number and unit of measurement
☐	☒	A statement on whether measurements were taken from distinct samples or whether the same sample was measured repeatedly
☐	☒	The statistical test(s) used AND whether they are one- or two-sided<br>*Only common tests should be described solely by name; describe more complex techniques in the Methods section.*
☐	☒	A description of all covariates tested
☐	☒	A description of any assumptions or corrections, such as tests of normality and adjustment for multiple comparisons
☐	☒	A full description of the statistical parameters including central tendency (e.g. means) or other basic estimates (e.g. regression coefficient) AND variation (e.g. standard deviation) or associated estimates of uncertainty (e.g. confidence intervals)
☐	☒	For null hypothesis testing, the test statistic (e.g. *F*, *t*, *r*) with confidence intervals, effect sizes, degrees of freedom and *P* value noted<br>*Give P values as exact values whenever suitable.*
☒	☐	For Bayesian analysis, information on the choice of priors and Markov chain Monte Carlo settings
☒	☐	For hierarchical and complex designs, identification of the appropriate level for tests and full reporting of outcomes
☐	☒	Estimates of effect sizes (e.g. Cohen's *d*, Pearson's *r*), indicating how they were calculated

Our web collection on statistics for biologists contains articles on many of the points above.

Software and code

Policy information about availability of computer code

Data collection	Database searching was conducted in proteome discoverer 2.2 (Thermo Scientific) using the Mascot search engine (Matric Science), searching the Swissprot human reference proteome (Uniprot, downloaded April 2018)
Data analysis	MS2 spectra that gave rise to a peptide spectral match were searched against MS2 spectra arising from parent ions with a mass shift corresponding to heavy/light labeling (i.e. ~4 Da shift) using an in-house script. Briefly, mass spectrometry files were converted to mzXML using default setting in MSConvert2 and read into R Version 4.3.2 using the mzR package. For each MS2 with a PSM (from Mascot database searching), peak lists were extracted for MS2 scans arising from parent ions with a 4Da shift. Peak lists were exported and compared for matching and complementary ions as described for HiLight-PTM (Whitwell 2019), using a tolerance of 0.5 and 0.8 mz for matching the same and complementary-mass shift ions respectively. Pearson's product moment correlation coefficient was then calculated in R for the intensity of the matched and complementary mass-shifted ions.

For manuscripts utilizing custom algorithms or software that are central to the research but not yet described in published literature, software must be made available to editors and reviewers. We strongly encourage code deposition in a community repository (e.g. GitHub). See the Nature Portfolio guidelines for submitting code & software for further information.

Data

Policy information about availability of data

All manuscripts must include a data availability statement. This statement should provide the following information, where applicable:

- Accession codes, unique identifiers, or web links for publicly available datasets
- A description of any restrictions on data availability
- For clinical datasets or third party data, please ensure that the statement adheres to our policy

Analytical spectra for 7 and 8 and further data are provided in the SI. Raw data is available upon request from the authors. The mass spectrometry proteomics data have been deposited to the ProteomeXchange Consortium via the PRIDE partner repository with the dataset identifier PXD052790.

Research involving human participants, their data, or biological material

Policy information about studies with human participants or human data. See also policy information about sex, gender (identity/presentation), and sexual orientation and race, ethnicity and racism.

Reporting on sex and gender	The research did not include studies with human participants or human data.
Reporting on race, ethnicity, or other socially relevant groupings	The research did not include studies with human participants or human data.
Population characteristics	The research did not include studies with human participants or human data.
Recruitment	The research did not include studies with human participants or human data.
Ethics oversight	The research did not include studies with human participants or human data.

Note that full information on the approval of the study protocol must also be provided in the manuscript.

Field-specific reporting

Please select the one below that is the best fit for your research. If you are not sure, read the appropriate sections before making your selection.

☒ Life sciences ☐ Behavioural & social sciences ☐ Ecological, evolutionary & environmental sciences

For a reference copy of the document with all sections, see nature.com/documents/nr-reporting-summary-flat.pdf

Life sciences study design

All studies must disclose on these points even when the disclosure is negative.

Sample size	In vitro enzymatic reactions were performed in triplicate to permit statistical comparison where necessary. In most cases, a binary (yes/no) answer was sufficient - i.e. does the enzyme react with the co-factor in the experimental conditions. Cell line work was performed N=1, while performing across a gradient of SAM and BrDeazaSAM. This aim of this experiment was identification of DOT1L-substrates without focus on a quantitative assessment.
Data exclusions	No data excluded.
Replication	In vivo experiments were not replicated, however validation of putative targets was performed, as described in the manuscript.
Randomization	Not applicable.
Blinding	Not applicable.

Reporting for specific materials, systems and methods

We require information from authors about some types of materials, experimental systems and methods used in many studies. Here, indicate whether each material, system or method listed is relevant to your study. If you are not sure if a list item applies to your research, read the appropriate section before selecting a response.

Materials & experimental systems

n/a	Involved in the study
☒	☐ Antibodies
☐	☒ Eukaryotic cell lines
☒	☐ Palaeontology and archaeology
☒	☐ Animals and other organisms
☒	☐ Clinical data
☒	☐ Dual use research of concern
☒	☐ Plants

Methods

n/a	Involved in the study
☒	☐ ChIP-seq
☒	☐ Flow cytometry
☒	☐ MRI-based neuroimaging

Eukaryotic cell lines

Policy information about cell lines and Sex and Gender in Research

Cell line source(s)	HEK293T (ACSS)
Authentication	Cell line was not autenticated.
Mycoplasma contamination	Cell lines within the cell-culture facility were screened for mycoplasma which was not detected.
Commonly misidentified lines (See ICLAC register)	NA

Plants

Seed stocks	*Report on the source of all seed stocks or other plant material used. If applicable, state the seed stock centre and catalogue number. If plant specimens were collected from the field, describe the collection location, date and sampling procedures.*
Novel plant genotypes	*Describe the methods by which all novel plant genotypes were produced. This includes those generated by transgenic approaches, gene editing, chemical/radiation-based mutagenesis and hybridization. For transgenic lines, describe the transformation method, the number of independent lines analyzed and the generation upon which experiments were performed. For gene-edited lines, describe the editor used, the endogenous sequence targeted for editing, the targeting guide RNA sequence (if applicable) and how the editor was applied.*
Authentication	*Describe any authentication procedures for each seed stock used or novel genotype generated. Describe any experiments used to assess the effect of a mutation and, where applicable, how potential secondary effects (e.g. second site T-DNA insertions, mosiacism, off-target gene editing) were examined.*
